# Defect-Mediated Threshold Voltage Tuning in β-Ga_2_O_3_ MOSFETs via Fluorine Plasma Treatment

**DOI:** 10.3390/nano15241896

**Published:** 2025-12-17

**Authors:** Lisheng Wang, Yifan Zhang, Junxing Dong, Jingzhuo Wang, Zenan Wang, Yuan Feng, Xianghu Wang, Si Shen, Hai Zhu

**Affiliations:** 1 State Key Laboratory of Optoelectronic Materials and Technologies, School of Physics, Sun Yat-Sen University, Guangzhou 510275, China; wanglsh23@mail2.sysu.edu.cn (L.W.); zhangyf93@mail2.sysu.edu.cn (Y.Z.); dongjx3@mail2.sysu.edu.cn (J.D.); wangjzh23@mail2.sysu.edu.cn (J.W.); wangzn8@mail2.sysu.edu.cn (Z.W.); fengy96@mail2.sysu.edu.cn (Y.F.); 2 School of Arts and Sciences, Shanghai Dianji University, Shanghai 200245, China

**Keywords:** β-Ga_2_O_3_, MOSFETs, heteroepitaxy, fluorine plasma, surface treatment

## Abstract

We demonstrate high-performance MOSFETs on β-Ga_2_O_3_ films grown by plasma-assisted molecular beam epitaxy (PA-MBE). The high crystalline quality of the β-Ga_2_O_3_ epilayer was confirmed by X-ray diffraction and atomic force microscopy. An optimized CF_4_-plasma treatment was employed to introduce fluorine (F) into the near-surface region, effectively suppressing donor-like states. The resulting devices exhibit an ultralow off-state current of 1 × 10^−9^ mA/mm and a stable on/off ratio of 10^5^. A controllable positive threshold voltage shift up to +12.4 V was achieved by adjusting the plasma duration. X-ray photoelectron spectroscopy indicates that incorporated F atoms form F–Ga-related bonds and compensate oxygen-related donor defects. Sentaurus TCAD simulations reveal reduced near-surface charge and a widened depletion region, providing a physical explanation for the experimentally observed increase in breakdown voltage from 453 V to 859 V. These results clarify the role of fluorine in modulating surface defect states in PA-MBE β-Ga_2_O_3_ and demonstrate an effective route for threshold-voltage engineering and leakage suppression in Ga_2_O_3_ power devices.

## 1. Introduction

With the continuous advancement of power-device scaling driven by demands for high power density and miniaturization, silicon (Si)-based power devices are confronting fundamental physical limitations. The relatively low critical electric field of Si (≤0.3 MV/cm) restricts further reduction in device dimensions while maintaining breakdown robustness, motivating the development of wide-bandgap semiconductor technologies [1,2,3]. Materials such as GaN, SiC, and Ga_2_O_3_ offer distinct advantages in this regard [4]. These materials exhibit superior Baliga’s figure of merit (BFOM), enabling the development of smaller, faster, more efficient, and reliable electronic devices [5,6].

Among these materials, β-Ga_2_O_3_ has attracted considerable attention due to its ultrawide bandgap (4.9 eV), extremely high critical breakdown field (8 MV/cm), and exceptional theoretical BFOM (3200) [7,8,9]. To improve the electrical performance of β-Ga_2_O_3_ MOSFETs, various device and material engineering techniques have been investigated, including field-plate structures, trench gate designs, ion implantation, and heterojunction engineering [10,11,12]. Post-growth surface treatments represent another effective approach for modulating interface states and improving device performance [13,14,15].

Fluorine-based treatments in particular have been widely studied. Prior reports have demonstrated that fluorine incorporation can deplete channel carriers and tune the threshold voltage in various semiconductor platforms, such as AlGaN/GaN high-electron-mobility transistors (HEMTs), amorphous InGaZnO (a-IGZO) thin-film transistors, and β-Ga_2_O_3_ MOSFETs [16,17,18]. Related studies on fluorine-treated Schottky diodes and edge terminations have shown leakage current reduction and breakdown voltage improvement through defect control and electric field redistribution [19,20,21]. Meanwhile, both theoretical and experimental studies have demonstrated that fluorine may act as a shallow donor and increase the carrier concentration in Ga_2_O_3_ films grown by different methods and in different crystal phases [22,23,24,25]. These diverse observations highlight the complex and system-dependent role of fluorine in β-Ga_2_O_3_, underscoring the need for a more detailed understanding of F-induced surface and interface modifications.

In this work, we apply fluorine plasma surface engineering to plasma-assisted molecular beam epitaxy (PA-MBE) grown β-Ga_2_O_3_ films on 2-inch sapphire substrates and demonstrate its simultaneous impact on threshold voltage tuning and breakdown enhancement in lateral MOSFETs. The F treatment effectively reduces near-surface donor-like states, enabling an ultralow $I_{off}$ of 1 × 10^−9^ mA/mm and a controllable positive $V_{th}$ shift up to +12.4 V by adjusting the plasma duration. XPS analysis reveals F incorporation and the formation of F–Ga-related bonding, while TCAD simulations suggest that the suppressed near-surface charge and the widened depletion region are responsible for the experimentally observed increase in breakdown voltage. These results clarify the role of fluorine in modifying surface defect states in PA-MBE β-Ga_2_O_3_ and provide an effective route for engineering threshold voltage and leakage in Ga_2_O_3_-based power devices.

## 2. Experimental Section

### 2.1. Growth of β-Ga_2_O_3_ Thin Films

The β-phase Ga_2_O_3_ films were grown using PA-MBE (SVT-35, SVT Associates, Eden Prairie, MN, USA). Firstly, the cleaned sapphire substrates were degassed in the buffer chamber at 170 °C for 4 h to remove residual moisture and surface contaminants. The substrates were then transferred into the growth chamber and annealed at 750 °C under an oxygen-rich atmosphere for 15 min to further desorb surface impurities. Subsequently, β-Ga_2_O_3_ films were grown for 2 h at 700 °C with a Ga source temperature of 950 °C and an oxygen flow of 1.6 sccm. The O-to-Ga flux ratio was maintained above the stoichiometric value to ensure oxygen-rich growth conditions. Such conditions have been demonstrated to suppress suboxide (Ga_2_O) formation and GaO_x_ decomposition, leading to improved growth rate and crystalline quality of films [26,27]. The resulting β-Ga_2_O_3_ films exhibited high crystalline quality with an average thickness of ~300 nm, as confirmed by subsequent structural characterization.

### 2.2. Device Fabrication

The fabrication of β-Ga_2_O_3_ circular transmission line model (CTLM) and MOSFET structures was carried out using high-resolution ultraviolet lithography. Prior to device fabrication, the as-grown β-Ga_2_O_3_ films were cleaned by sequential ultrasonic treatment in acetone, ethanol, and deionized water (5 min each) to remove surface organic contaminants. F-plasma treatment was conducted in an inductively coupled plasma reactive ion etching (ICP-RIE) chamber using CF_4_/O_2_ (20/5 sccm) at 5.0 mTorr, with an ICP coil power of 200 W and an RF platen power of 100 W at a chuck temperature of 300 K. The treatment was applied across the entire wafer area. The treatment duration was varied to investigate the time-dependent effects. For MOSFETs fabrication, a 120 nm thick SiO_2_ gate dielectric was subsequently deposited by ICP chemical vapor deposition (ICP-CVD) using N_2_O/SiH_4_ gas flows of 20/5 sccm at 2.0 mTorr. The deposition was carried out at an ICP power of 1000 W, an RF power of 20 W, and a DC bias of 80 V at 575 K. No post-deposition annealing was performed. The source/drain (S/D) contact windows were subsequently defined by maskless lithography and opened using RIE. Ti/Au (10/100 nm) S/D electrodes were then formed through thermal evaporation followed by a lift-off process. Finally, the Ni/Au (30/100 nm) gate (G) electrodes were deposited using the same lithography and lift-off procedures.

Each β-Ga_2_O_3_ MOSFET featured a circular geometry, with the source electrode radius designed as 200 μm. The gate adopted an annular configuration with inner and outer radii of 210 μm and 220 μm, respectively, corresponding to a gate length ($L_{G}$) of 10 μm and an equivalent gate-source spacing ($L_{GS}$). The drain electrode was designed in a semi-annular layout with an inner radius of 240 μm, yielding a drain-source spacing ($L_{DS}$) of 40 μm. This symmetric configuration ensured uniform electric-field distribution and stable gate control across the active region.

### 2.3. Characterization

The morphology of the β-Ga_2_O_3_ films was examined using field-emission scanning electron microscopy (FE-SEM, S4800, Hitachi, Tokyo, Japan) and atomic force microscopy (AFM, nanoIR2-s, Anasys Instruments, Billerica, USA) to evaluate the surface topography and roughness. The crystalline structure and orientation were characterized by X-ray diffraction (XRD, Empyrean, Malvern Panalytical, Great Malvern, UK), while the chemical states and elemental composition were analyzed using X-ray photoelectron spectroscopy (XPS, Nexsa, Thermo Fisher Scientific, Waltham, MA, USA). The XPS measurements were performed with an Al Kα excitation source (hν = 1486.6 eV). The analyzer operated in constant analyzer energy (CAE) mode with a pass energy of 40 eV and a step size of 0.05 eV. Spectra were collected at a take-off angle of 90°. A dual-beam charge neutralization system, comprising low-energy electrons and low-energy ions, was employed to compensate for surface charging during the measurements.

All electrical measurements of the β-Ga_2_O_3_ MOSFETs were performed at room temperature (RT) under ambient conditions. The transfer and output characteristics were obtained using a semiconductor parameter analyzer (4200A-SCS, Keithley Instruments, Beaverton, OR, USA), and the breakdown voltage was determined with a power device analyzer (B1500A, Agilent Technologies, Santa Clara, CA, USA). Breakdown voltage measurements were conducted on multiple devices, and the values reported correspond to representative characteristics that reflect the consistent behavior observed among the tested samples under the same measurement conditions.

### 2.4. XPS Analysis

All XPS spectra were charge-corrected to the C 1s peak at 284.8 eV. Peak fitting was performed using XPS Peak software (ver. 4.1, Cabit Information Technology, Shanghai, China), while the peak identification and the charge correction were verified using Thermo Avantage software (ver. 5.9921, Thermo Fisher Scientific, Waltham, USA). Standard fitting constraints commonly applied to metal-oxide systems were used. Mixed Gaussian-Lorentzian (GL(20)) line shapes were applied to all components. Binding energies were initialized from literature values and allowed to vary within ±0.2–0.3 eV. The FWHM was constrained to 1.0–2.0 eV, with differences between related chemical states limited to <0.3 eV.

## 3. Results and Discussion

**Figure 1:**
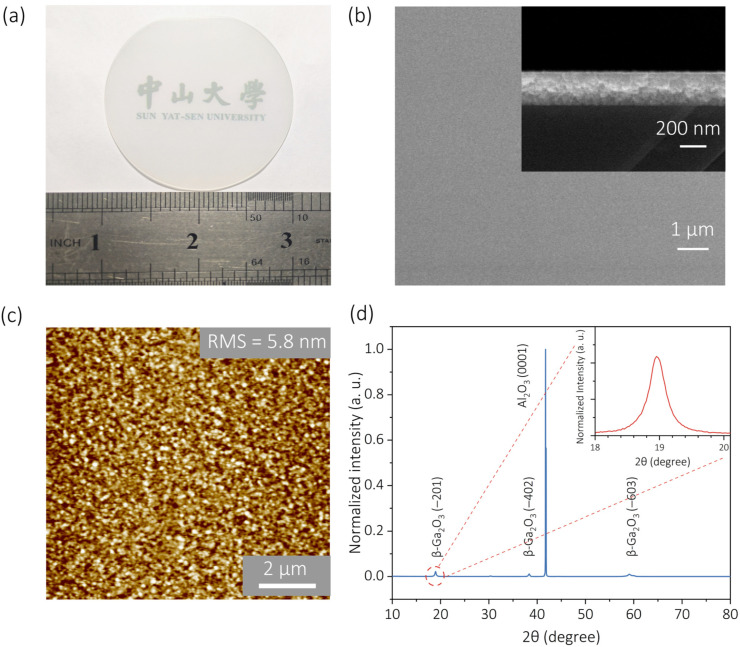
Optical image and crystalline properties of the β-Ga_2_O_3_ film grown on a 2-inch sapphire substrate via plasma-assisted molecular beam epitaxy (PA-MBE). (**a**) Optical image of the as-grown 2-inch β-Ga_2_O_3_ thin film wafer. (**b**) Scanning electron microscopy (SEM) image of the single-crystalline β-Ga_2_O_3_ film obtained by heteroepitaxy. Inset: cross-sectional SEM image showing a film thickness of approximately 300 nm. (**c**) Atomic force microscopy (AFM) image of the film surface with a root-mean-square (RMS) roughness of 5.8 nm. (**d**) X-ray diffraction (XRD) pattern of the β-Ga_2_O_3_ film on sapphire substrate. The three characteristic peaks located at 18.9°, 38.4° and 59.0° are related to the (−201), (−402) and (−603) lattice planes of β-Ga_2_O_3_, respectively. Inset: enlarged view of the (−201) peak, with a full width at half maximum (FWHM) of 0.31°, confirming the high crystallinity of the β-Ga_2_O_3_ film.

The as-grown 2-inch β-Ga_2_O_3_ wafer exhibited a transparent and optically uniform film surface on the c-plane sapphire substrate, as shown in Figure 1a. AFM and planar-view SEM images revealed a smooth surface morphology with a root-mean-square (RMS) roughness of 5.8 nm over a 10 × 10 μm^2^ area (Figure 1b,c). The cross-sectional SEM image further confirmed a uniform film thickness of approximately 300 nm (inset in Figure 1b). XRD analysis was conducted to evaluate the crystalline quality of the β-Ga_2_O_3_ film (Figure 1d). Distinct diffraction peaks located at 18.9°, 38.4°, and 59.0° correspond to the (−201), (−402), and (−603) planes of β-Ga_2_O_3_, respectively [28]. The absence of any additional peaks indicated a single-phase β-Ga_2_O_3_ epilayer with well-defined crystallographic orientation. Collectively, the excellent optical uniformity, smooth surface morphology, and phase-pure crystal structure verified by XRD confirmed the successful heteroepitaxial growth of a high-quality β-Ga_2_O_3_ thin film on sapphire substrate via PA-MBE.

**Figure 2:**
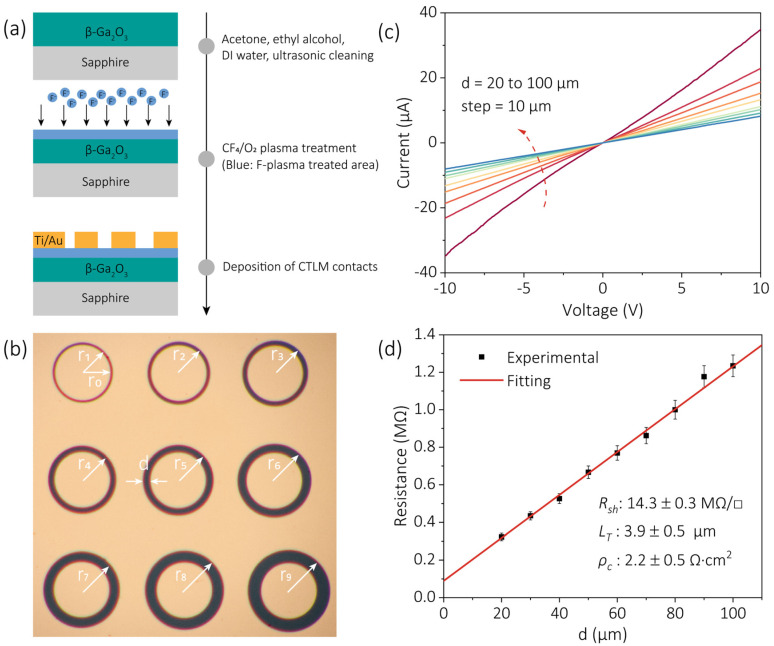
Fabrication process, optical micrograph, and I–V characteristics of fabricated β-Ga_2_O_3_ circular transmission line models (CTLMs). (**a**) Cross-sectional schematic and fabrication flow of a β-Ga_2_O_3_ CTLM. (**b**) Optical micrograph of the fabricated CTLM structures with a fixed inner-circle radius ($r_{0}$) of 200 μm. The outer-circle radii ($r_{i},\;i=1\sim 9$) range from 220 to 300 μm in steps of 10 μm, where d is defined as $r_{i}-r_{0}$. (**c**) I–V curves of β-Ga_2_O_3_ CTLMs subjected to a 3 min surface treatment. (**d**) Extraction of sheet resistance and contact resistance from the I–V data.

The schematic structure and fabrication process of the β-Ga_2_O_3_ circular transmission line model (CTLM) patterns are illustrated in Figure 2a. The optical microscopy image of the fabricated CTLM structures is shown in Figure 2b, where each device featured a fixed inner-circle radius ($r_{0}$ = 200 μm). The outer-circle radii ($r_{i},\;i=1\sim 9$) varied from 220 to 300 μm in 10 μm increments, defining the electrode gap spacing $d=r_{i}-r_{0}$ (20 to 100 μm, in steps of 10 μm). Figure 2c presents the current–voltage (I–V) characteristics of CTLMs fabricated on β-Ga_2_O_3_ films subjected to a 3 min F-plasma treatment. For comparison, additional I–V curves corresponding to 5 min and 7 min treatments are provided in Supplementary Material, Figure S1. In contrast to the as-grown films (Supplementary Material, Figure S2), the S/D contacts formed on F-plasma treated β-Ga_2_O_3_ exhibited clear ohmic behavior, indicating a substantial reduction in contact barrier height and improved carrier injection at the metal/semiconductor interface. This improvement can be attributed to the fluorine-passivation effect, which effectively reduced surface states and improved the interfacial quality between the metal and β-Ga_2_O_3_. As further supported by AFM measurements (Supplementary Material, Figure S3), the surface roughness of β-Ga_2_O_3_ continuously decreased with increasing plasma treatment time, indicating smoother morphology and reduced surface contamination. The smoother surface mitigated impurity scattering and consequently enhanced carrier mobility. To exclude the possibility of plasma-induced structural degradation, the F-plasma etching rate was also evaluated and found to be approximately 5.4 nm/min (Supplementary Material, Figure S4). Given the 300 nm thickness of the β-Ga_2_O_3_ layer, this slow etching rate ensured that the plasma treatment exerted a negligible impact on the overall film thickness and structural integrity.

The total resistance ($R_{T}$) as a function of d exhibited a clear linear dependence (Figure 2d), consistent with the behavior expected from the CTLM structure. The relationship can be expressed as [29]:

(1) $$R_{T}=\frac{R_{sh}}{2\pi r_{0}}\left(d+{2L}_{T}\right)$$

where $R_{sh}$ represents the sheet resistance and $L_{T}$ is the transfer length. From the linear fitting, $R_{sh}$ and $L_{T}$ were extracted to be 14.3 MΩ/square and 3.9 μm, respectively. The specific contact resistivity ($\rho_{c}$) was subsequently calculated according to [30]:

(2) $$\rho_{c}=R_{sh}L_{T}^{2}$$

resulting in a value of 2.2 Ω·cm^2^. This relatively high $\rho_{c}$ value, compared with previously reported values, can be primarily attributed to the unintentional doping level of the Ga_2_O_3_ thin film, which limits carrier concentration near the contact interface and thereby increases contact resistance.

**Figure 3:**
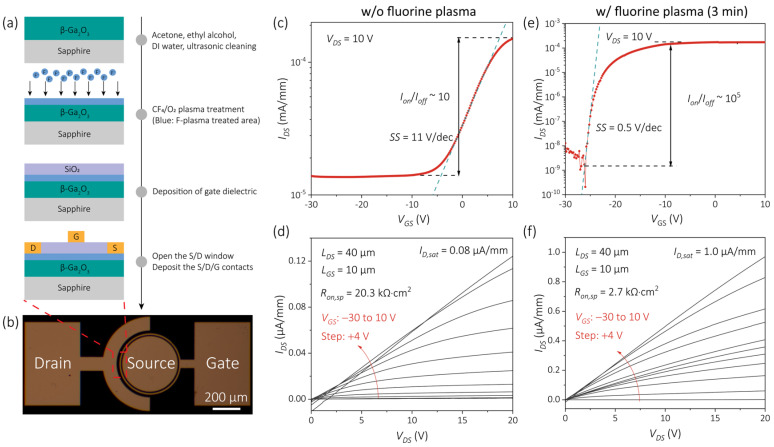
Fabrication process, optical micrograph, and I–V characteristics of β-Ga_2_O_3_ MOSFETs. (**a**) Cross-sectional schematic illustrating the β-Ga_2_O_3_ MOSFET structure and fabrication flow. (**b**) Optical micrograph of a representative β-Ga_2_O_3_ MOSFET. The radius of the circular source electrode is 200 μm, while the inner and outer radii of annular gate electrode are 210 μm and 220 μm, resulting in both a gate-to-source ($L_{GS}$) and a gate length ($L_{G}$) of 10 μm. The drain-to-source ($L_{DS}$) is designed to be 40 μm. (**c**) Typical transfer characteristics of the untreated device (F0) at $V_{DS}$ = 10 V. (**d**) Output characteristics of F0 under various applied $V_{GS}$. (**e**) Transfer characteristics of the 3 min fluorine-treated device (F3) at $V_{DS}$ = 10 V. (**f**) Output characteristics of F3 measured under various applied $V_{GS}$.

The fabrication flow of the β-Ga_2_O_3_ MOSFETs is summarized in Figure 3a, and an optical micrograph of a representative device is shown in Figure 3b. A circular source electrode was adopted to alleviate electric-field crowding commonly observed in rectangular layouts, while an annular gate electrode was employed to suppress lateral electric-field spreading and ensure electrical isolation from adjacent structures. For clarity, MOSFETs without F-plasma treatment are denoted as F0, while devices subjected to 3, 5, 7 min treatments are referred to as F3, F5, and F7, respectively.

The transfer characteristics of F0 and F3 under a drain bias ($V_{DS}$) of 10 V are presented in Figure 3c,e. The untreated device (F0) exhibited a relatively large off-state current ($I_{off}$) of ~1 × 10^−5^ mA/mm, resulting in a limited on/off current ratio ($I_{on}$/$I_{off}$) of ~10^1^. In contrast, F3 demonstrated clear gate-voltage modulation with an $I_{off}$ suppressed to 1 × 10^−9^ mA/mm. The $I_{off}$ was reduced by nearly four orders of magnitude, leading to an $I_{on}$/$I_{off}$ exceeding 10^5^. The subthreshold swing (SS) was extracted to be 0.50 V/dec according to [31]:

(3) $$SS=\;{\left(\frac{d\;log(I_{DS})}{dV_{GS}}\right)}^{-1}$$

where $I_{DS}$ and $V_{GS}$ represent the drain-to-source current and gate-to-source voltage, respectively. The relatively small SS value indicates efficient gate electrostatic control and a reduced interfacial trap density, consistent with the surface passivation effect induced by F incorporation.

The output characteristics of F0 and F3 are shown in Figure 3d,f. For F0, both the saturation current ($I_{D,sat}$) and linear region were severely limited, and a noticeable negative current appeared at low drain biases ($V_{DS}$ < 5 V), which may arise from drain-to-gate electron transport through defective interface states. By comparison, F3 exhibited typical field-effect transistor behavior, with linear $I_{DS}$–$V_{DS}$ curves and minimal current crowding at $V_{DS}$ < 5 V, confirming the improved ohmic contact achieved after F-plasma treatment.

**Figure 4:**
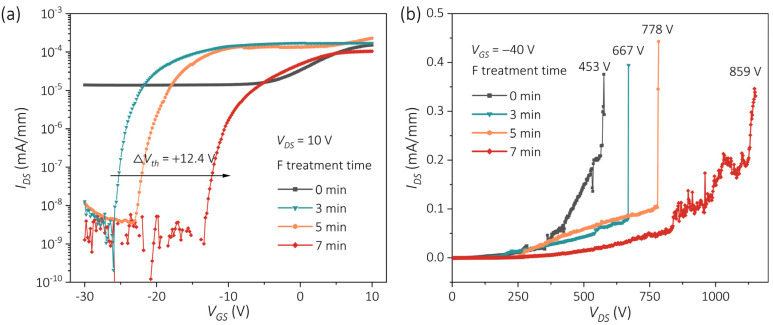
(**a**) Transfer characteristics of β-Ga_2_O_3_ MOSFETs after different F-plasma treatment durations. The transfer curves exhibit a positive threshold voltage shift of +12.4 V. (**b**) Breakdown characteristics of devices with varying treatment times. The breakdown voltage increases from 453 V (F0) to 859 V (F7).

The influence of F-plasma treatment duration on the transfer and breakdown characteristics of β-Ga_2_O_3_ MOSFETs was systematically examined to clarify the role of F-induced surface modification. As shown in Figure 4a, the $I_{off}$ remained consistently at the ultralow level of 10^−9^ mA/mm with increasing treatment time, demonstrating the robustness of the surface passivation. It is noted that such a low current is close to the measurement limit of the parameter analyzer; therefore, the slight fluctuations observed in $I_{off}$ are attributed to instrument-induced noise.

Meanwhile, the $V_{th}$ exhibited a monotonic positive shift with increasing F-plasma treatment time, which can be attributed to the progressive charge compensation at the SiO_2_ interface and the depletion of near-surface donor-like defects. Despite the significant $V_{th}$ shift, the devices have not yet achieved true enhancement-mode (E-mode) operation, implying that further improvements in epitaxial quality and device architecture will be required to achieve reliable E-mode behavior.

In addition, the dependence of the breakdown voltage ($V_{br}$) on F-plasma treatment duration is summarized in Figure 4b. For the unintentionally doped β-Ga_2_O_3_ films used in this study, $V_{br}$ was defined as the $V_{DS}$ at which the $I_{DS}$ reached 0.1 mA/mm. The measured $V_{br}$ increased significantly from 453 V (F0) to 859 V (F7), representing a nearly twofold enhancement. This improvement in blocking capability is attributed to the widening of the depletion region and the reduction of leakage-related defect states at the β-Ga_2_O_3_/SiO_2_ interface induced by optimized F incorporation. A qualitative assessment of device robustness was also performed (Supplementary Material, Figure S4). The fluorinated devices maintained stable $I_{off}$ and $I_{on}$/$I_{off}$ for up to 90 days in air, with only small $V_{th}$ variations, while more detailed trends are discussed in the Supplementary Material.

Table 1 summarizes the extracted electrical parameters of β-Ga_2_O_3_ MOSFETs with different F-plasma treatment durations, highlighting the pronounced improvements in subthreshold swing, threshold voltage stability, off-state leakage, and breakdown voltage. These results collectively confirm that controlled F incorporation effectively passivates donor-like surface defects and improves the interface quality, leading to enhanced device performance.

**Figure 5:**
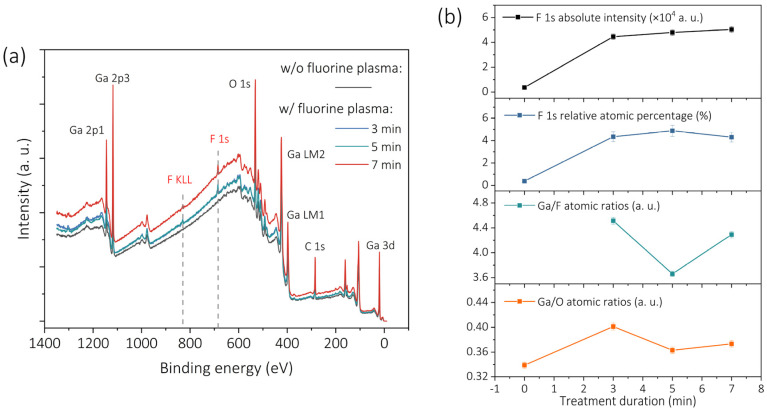
(**a**) X-ray photoelectron spectroscopy (XPS) survey spectra of the pristine and fluorinated β-Ga_2_O_3_ surfaces, revealing the emergence of the F 1s and F KLL peaks after F-plasma treatment. (**b**) Time-dependent evolution of the absolute F 1s intensity, the relative F 1s atomic percentage, and the Ga/F and Ga/O atomic ratios for samples subjected to F-plasma treatment.

To elucidate the chemical states and bonding configurations induced by F-plasma treatment, comprehensive XPS measurements were performed on β-Ga_2_O_3_ films. All spectra were calibrated to the adventitious C 1s peak at 284.8 eV [32,33] (Supplementary Material, Figure S5). The survey spectra revealed a distinct F 1s peak at 686.1 eV and an F KLL peak at 833 eV in the fluorinated samples, confirming successful fluorine incorporation (Figure 5a) [34]. Figure 5b summarizes the evolution of F incorporation, including the F 1s peak intensity and its atomic percentage. While the absolute F 1s intensity increases monotonically with exposure time, the F atomic fraction reaches a small maximum at an intermediate exposure duration (5 min) and decreases slightly after prolonged treatment (7 min). Figure 5b further presents the corresponding Ga/O and Ga/F ratios, both of which vary inversely with the F atomic fraction. As F atomic percentage increases, Ga/O and Ga/F decrease accordingly. This behavior indicates that fluorine substitutes a portion of lattice oxygen or occupies oxygen-vacancy sites [35], effectively altering the local chemical environment. As for the slight reduction in the F atomic fraction at longer exposure, it may originate from plasma-induced surface etching or from increased adsorption of –OH groups that partially dilute the detected F concentration. These possibilities require further investigation.

**Figure 6:**
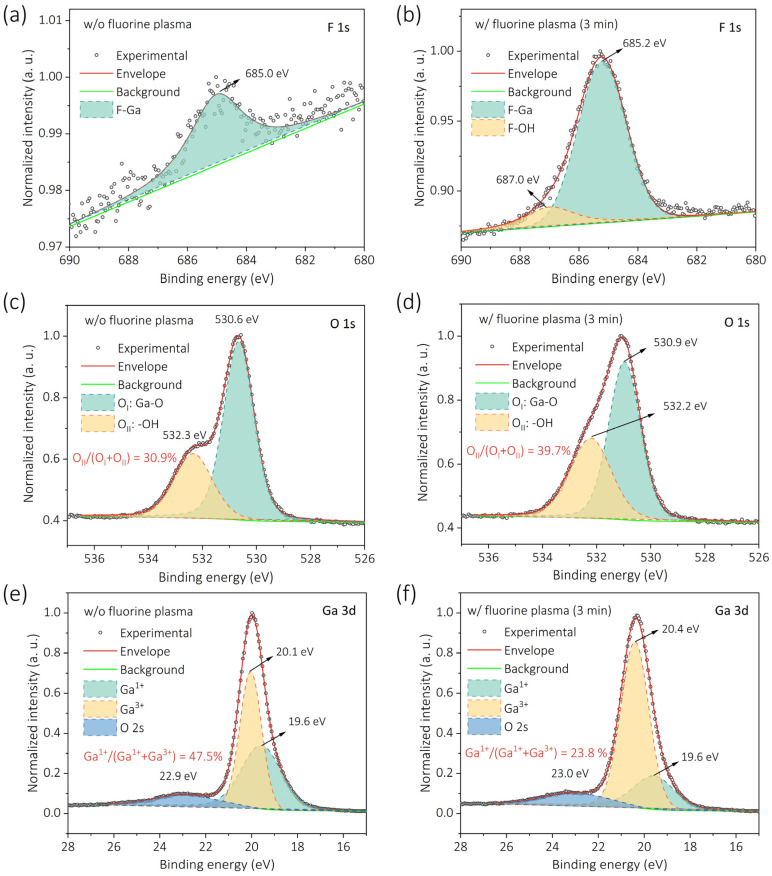
(**a**) F 1s spectrum of the pristine sample (**b**) F 1s spectrum of the 3 min treated sample, showing two peaks: F–Ga bonds (685.2 eV) and F–OH interactions (687.0 eV). (**c**,**d**) O 1s spectra of the pristine and 3 min treated samples, deconvoluted into O_I_ (O–Ga bonds, 530.6 eV) and O_II_ (surface –OH groups, 532.3 eV). (**e**,**f**) Ga 3d spectra of the pristine and 3 min treated samples, deconvoluted into Ga^1+^ (19.5 eV) and Ga^3+^ (20.4 eV).

To clarify the detailed chemical states associated with fluorine incorporation, we next examine the high-resolution F 1s, O 1s, Ga 3d, and Ga 2p spectra. As shown in Figure 6a, the pristine β-Ga_2_O_3_ film exhibits only a barely detectable F 1s signal near 685.0 eV. After a 3 min exposure (Figure 6b), the F 1s peak becomes prominent and can be deconvoluted into two components centered at 685.2 (F–Ga bonds) and 687.0 eV (F–OH interactions), respectively [36]. This observation indicates simultaneous fluorine incorporation into the lattice and modification of the surface hydroxyl environment.

Consistent trends are observed in the O 1s spectra (Figure 6c,d). Both pristine and F-plasma treated samples show two components at ~530.6 and 532.3 eV, corresponding to O_I_ (Ga–O bonds) and O_II_ (surface –OH groups), respectively [36]. Following fluorination, the –OH-related peak becomes stronger, consistent with the enhanced –OH adsorption inferred from Figure 5. This increase likely results from the high electronegativity of F atoms that promote F–HO bond formation [37]. The Ga 3d spectra (Figure 6e,f) further corroborate the defect passivation behavior implied by the decreasing Ga/O and Ga/F ratios. In the untreated film, the Ga^1+^ component at 19.6 eV contributes significantly to the total signal. After F-plasma exposure, this Ga^1+^ component is substantially suppressed (47.5% to 23.8%), while Ga^3+^ peak near 20.4 eV (stoichiometric Ga_2_O_3_) becomes dominant [38]. The transition indicates that incorporated F atoms effectively passivate oxygen vacancies within the Ga_2_O_3_ lattice, consistent with the observed suppression of donor-like defect states [39]. Because potential Ga–F features cannot be resolved separately in the Ga 3d region, analysis of deeper core levels is required to distinguish Ga–F contributions.

**Figure 7:**
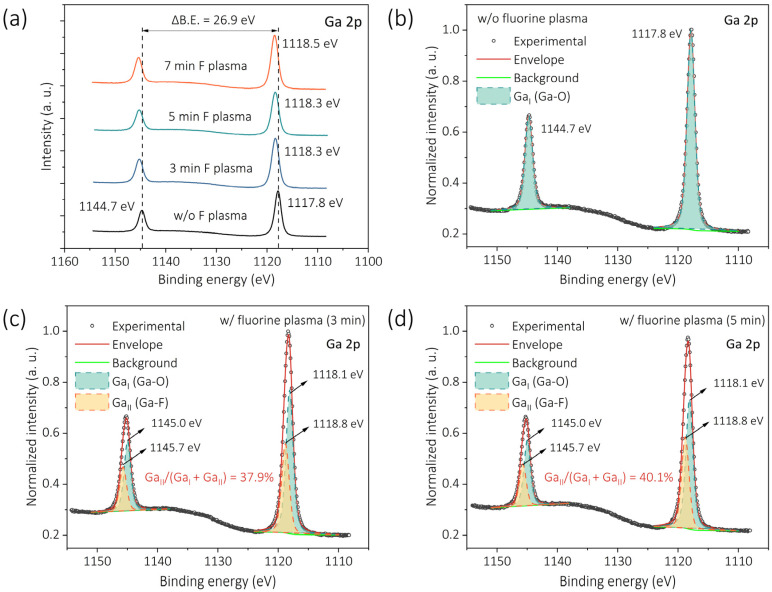
(**a**) Overview of the Ga 2p spectra for the untreated sample and the samples subjected to 3, 5, and 7 min F-plasma treatments. Component analysis of the Ga 2p spectrum for (**b**) the untreated sample, (**c**) the sample treated for 3 min, and (**d**) the sample treated for 5 min.

To further clarify the contribution of Ga–F bonding and assess the effective depth of fluorine incorporation, Ga 2p spectra were examined (Figure 7). As shown in Figure 7a, both the Ga 2p_3/2_ and Ga 2p_1/2_ peak positions gradually shift toward higher binding energies with increasing F-plasma treatment time. This observation is consistent with F incorporation, as F is known to induce pronounced chemical shifts in neighboring elements [40,41]. Deconvolution of the Ga 2p_3/2_ peak (Figure 7b–d) reveals a Ga–O component at ~1118.1 eV and a Ga–F component at ~1118.8 eV [42], whose relative fraction increases with plasma duration (from 37.9% to 40.1%). Given the larger probing depth of Ga 2p relative to Ga 3d, the emergence and growth of the Ga–F component indicate that F incorporation is not confined to the extreme surface but extends into the near-surface region of the β-Ga_2_O_3_ lattice.

Together with the elemental-ratio evolution shown in Figure 5b, these spectral features establish a consistent chemical picture: fluorine atoms substitute for oxygen or occupy vacancy sites, passivating donor-like defects while simultaneously modifying the surface hydroxyl environment. This multichannel chemical evolution directly underpins the enhanced electrical performance observed in fluorinated β-Ga_2_O_3_ MOSFETs.

To further elucidate the charge-modulation mechanism in β-Ga_2_O_3_ MOSFETs, Sentaurus TCAD simulations were carried out based on the experimentally derived device geometry (Supplementary Material, Figure S6). Donor-like trap states associated with oxygen-vacancy-related defects were introduced into the model, with their spatial distribution adjusted to reflect the experimentally observed higher defect density near the surface region. It should be noted that the specific values used in the simulation were chosen solely for qualitative analysis, and the simulation outcomes were robust against reasonable variations in these parameters.

**Figure 8:**
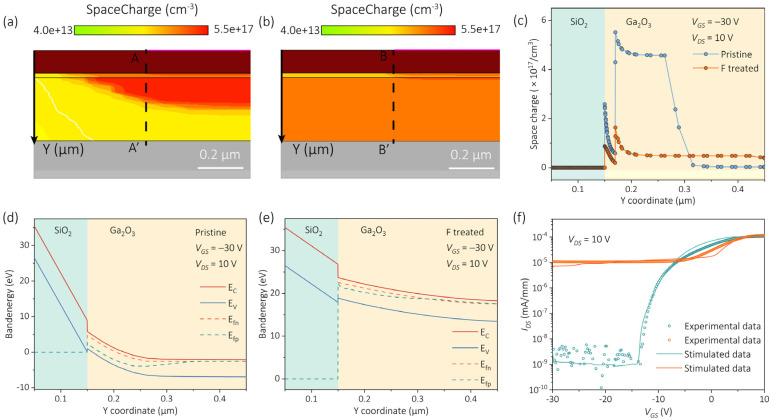
TCAD-simulated analysis of the β-Ga_2_O_3_ MOSFETs. (**a**,**b**) Space charge distributions at $V_{GS}\;$ = −30 V and $V_{DS}\;$ = 10 V for the pristine and the F-plasma treated devices. (**c**) Cross-sectional profiles of the space charge along the AA’ and BB’ cutlines. (**d**,**e**) Energy-band diagrams extracted along AA’ and BB’. (**f**) Transfer characteristics: TCAD simulations (solid lines) versus experimental data (open circles); Green: the F-plasma treated device, orange: the pristine device.

The simulated space-charge distributions under a bias condition of $V_{GS}$ = −30 V and $V_{DS}$ = 10 V revealed distinct differences between the pristine and F-treated devices (Figure 8a,b). Cross-sectional charge density profiles extracted along the AA’ and BB’ cutlines (dashed lines in Figure 8a,b) demonstrated a substantial reduction in peak charge density after fluorine incorporation (Figure 8c), indicating effective neutralization of donor-like defects and suppression of interfacial charge trapping. The $V_{th}$ can be expressed as [43]:

(4) $$V_{th}=\psi_{bi}-\frac{qN_{i}d^{2}}{2\varepsilon_{s}}$$

where $\psi_{bi}$, $N_{i}$, q, d, and $\varepsilon_{s}$ denote the built-in potential, trap concentration, electronic charge, depletion width, and permittivity of the channel material, respectively. According to this relationship, the reduction of $N_{i}$ induced by F passivation leads to a positive shift in $V_{th}$. Energy band diagrams further illustrate these effects (Figure 8d,e): in the pristine device, the potential drop is primarily confined within the SiO_2_ dielectric, causing localized electric-field crowding and premature breakdown. In contrast, the F-plasma-treated device exhibits a broadened depletion region and a more uniform potential distribution across both SiO_2_ and β-Ga_2_O_3_ layers, effectively mitigating field localization and enhancing the $V_{br}$. Finally, the simulated transfer characteristics show excellent agreement with the experimental data (Figure 8f), validating the proposed charge-modulation and field-redistribution mechanism.

To facilitate a clearer understanding of the device performance, Table 2 summarizes representative parameters of β-Ga_2_O_3_ MOSFETs fabricated on both native and sapphire substrates. Overall, devices grown and processed on single-crystal native substrates typically exhibit superior performance, benefiting from higher film quality and lower defect densities. In contrast, MOSFETs on sapphire generally face inherent trade-offs due to heteroepitaxial constraints, which degrade material quality and limit electrical performance.

Compared with previously reported sapphire-based devices, our MOSFETs demonstrate an ultralow $I_{off}$ and achieve a notable $V_{br}$ even without employing field-plate structures. However, the presence of unintentional doping and residual defects restricts the on-state current and weakens gate electrostatic control, leading to a relatively modest $I_{on}$/$I_{off}$ and a larger SS. Further improvements in epitaxial film quality, intentional doping control, gate oxide engineering, and the incorporation of field-plate designs will be essential for pushing the device performance toward its full potential.

## 4. Conclusions

In conclusion, we successfully demonstrated β-Ga_2_O_3_ MOSFETs featuring ultralow off-state current and enhanced subthreshold characteristics through F-plasma treatment. The optimized devices achieved a low $I_{off}$ of 1 × 10^−9^ mA/mm and a positive $V_{th}$ shift of 12.4 V. Furthermore, the breakdown voltage was significantly improved from 453 V to 859 V. XPS analysis confirms that fluorine incorporation suppresses near-surface donor-like defects through the formation of Ga–F bonds and reduction of oxygen-vacancy-related states. Complementary TCAD simulations qualitatively reproduce the resulting charge-density reduction and electric-field redistribution, consistent with the experimentally observed positive $V_{th}$ shift and enhanced $V_{br}$. These findings not only provide fundamental insights into the mechanisms of fluorine incorporation in β-Ga_2_O_3_, but also establish a viable pathway for advancing the performance and reliability of Ga_2_O_3_-based power electronic devices.

## Figures and Tables

**Table 1:** Performance of β-Ga_2_O_3_ MOSFETs with varied F-plasma treatment durations.

Performance	F0	F3	F5	F7
$I_{on}/I_{off}$	10	10^5^	10^5^	10^5^
SS (V/dec)	11	0.50	0.66	0.63
$V_{th}$ (V)	-	−25.8	−22.8	−13.4
$V_{br}$ (V)	453	667	778	859

**Table 2:** Summary of some key parameters of β-Ga_2_O_3_ FETs fabricated on native and sapphire substrates.

$V_{br}$ (V)	$L_{DS}$ (μm)	$V_{th}$ (V)	$R_{on,sp}$ (mΩ·cm^2^)	$I_{DS,max}$ (mA/mm)	$I_{off}$ (mA/mm)	$I_{on}$ / $I_{off}$	SS (mV/dec)	Ref.
370	-	−15	-	39	10^−9^	10^10^	-	[44] *
2700	40	−12	78 kΩ·mm	0.2	10^−5^	10^5^	-	[11] *
800	11.4	−18	7.41	238.1	10^−9^	10^8^	86	[45]
400	40	−32	-	100	10^−9^	10^11^	210	[46]
-	70	−18	117.9 kΩ	10^−1^	10^−7^	10^6^	-	[25]
650	15	−8	-	10^−1^	10^−8^	10^7^	-	[47]
770	15	1.5	-	10^−4^	10^−8^	10^4^	-	
910	20	1.5	223	10^−3^	10^−7^	10^4^	-	[48]
240	20	1.5	752	0.7	10^−5^	10^5^	-	
1085	15	−7.5	600	3.71	10^−7^	10^7^	313	[49]
1250	15	−3.9	5820	0.5	10^−8^	10^7^	351	
859	40	−13.4	10^6^	10^−4^	10^−9^	10^5^	630	This work

*: β-Ga_2_O_3_ substrate.

## Data Availability

The original contributions presented in this study are included in the article/Supplementary Material. Further inquiries can be directed to the corresponding authors.

## Supplementary Materials

The following supporting information can be downloaded at: https://www.mdpi.com/article/10.3390/nano15241896/s1, Figure S1: (a,b) I–V characteristics of CTLMs fabricated on β-Ga_2_O_3_ films after 5-minute and 7-minute F-plasma treatments, respectively. (c,d) Extraction of sheet resistance and contact resistance from the corresponding I–V data; Figure S2: I–V curves of the CTLMs without F-plasma treatment; Figure S3: AFM images (10 × 10 μm^2^) and corresponding root-mean-square (RMS) surface roughness of β-Ga_2_O_3_ films: (a) as-grown, and after F-plasma treatment for (b) 3 min, (c) 5min, and (d) 7min; Figure S4: (a) AFM image of the etched step height after 7 min F-plasma treatment using a photoresist mask. (b) Height profile extracted along the dashed line AA’ in (a), showing a step height of approximately 38 nm, corresponding to an etch rate of ~5.4 nm/min; Figure S5: (a) Transfer characteristics of the β-Ga_2_O_3_ MOSFET (7-min F-plasma treated) measured after air exposure for 0, 90, and 180 days. (b) Extracted evolution of $V_{th}$, $I_{off}$, and $I_{on}$/$I_{off}$ as a function of storage time in air; Figure S6: C 1s XPS spectra for β-Ga_2_O_3_ films (a) without fluorine plasma, (b) with 3 minute, (c) 5 minute, (d) 7 minute fluorine plasma; Figure S7: (a) Overview of Ga 3d spectra and (b) Ga 2p for the untreated sample and samples subjected to 3, 5, and 7 min F-plasma treatments. Figure S8: Schematic cross-sectional view of the TCAD-simulated β-Ga_2_O_3_ MOSFET structure; Figure S9: (a), (b) Simulated channel electric-field distributions of MOSFETs without and with F-treatment at $V_{GS}\;$= −30 V and $V_{DS}\;$= 10 V, respectively. (c) Electric-field profiles extracted along the dashed lines indicated in (a) and (b). Table S1: Summary of XPS fitting parameters (values from representative fits reported in the manuscript).
