# Supplementary material for: Defect-Mediated Threshold Voltage Tuning in β-Ga2O3 MOSFETs via Fluorine Plasma Treatment
Source: Nanomaterials (Basel). 2025 Dec 17;15(24):1896. doi: 10.3390/nano15241896 (PMC12736358; doi:10.3390/nano15241896)
Supplement: Supplementary file 1 [file nanomaterials-15-01896-s001.zip › nanomaterials-4013748-supplementary.docx]

Supplementary material

**Defect-Mediated Threshold Voltage Tuning in β-Ga_2_O_3_ MOSFETs via Fluorine Plasma Treatment**

Lisheng Wang,^1^ Yifan Zhang,^1^ Junxing Dong,^1^ Jingzhuo Wang,^1^ Zenan Wang,^1^ Yuan Feng,^1^ Xianghu Wang,^2,*^ Si Shen,^2,*^ and Hai Zhu^1,*^

^1^State Key Laboratory of Optoelectronic Materials and Technologies, School of Physics, Sun Yat-Sen University, Guangzhou 510275, China

^2^School of Arts and Sciences, Shanghai Dianji University, Shanghai 200245, China

*E-mail: [wangxh@sdju.edu.cn,](mailto:wangxh@sdju.edu.cn.,) 32188@sdju.edu.cn, [zhuhai5@mail.sysu.edu.cn](mailto:zhuhai5@mail.sysu.edu.cn).


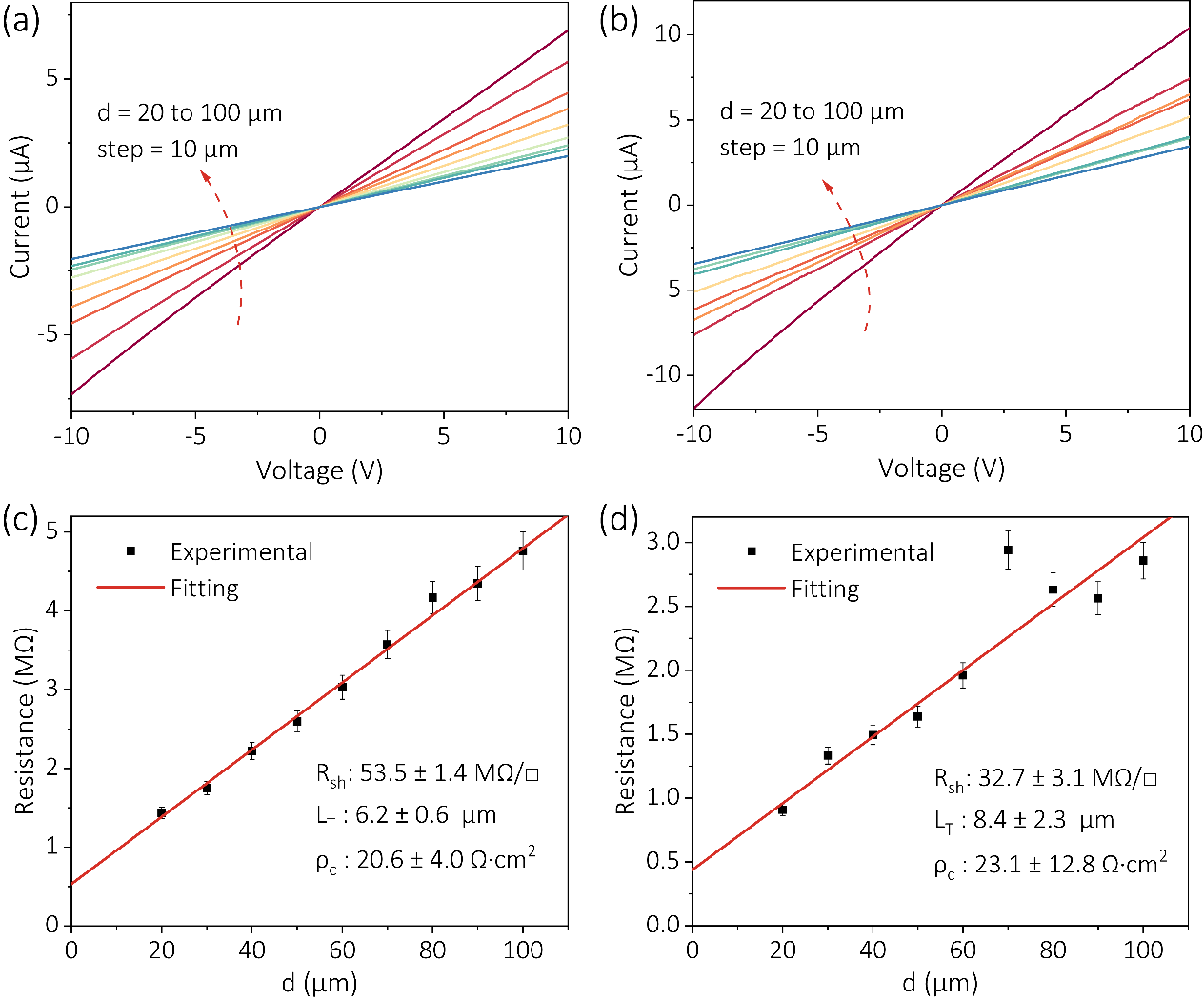


**Figure S1. (a, b)** I-V characteristics of CTLMs fabricated on β-Ga_2_O_3_ films after 5-minute and 7-minute F-plasma treatments, respectively. **(c, d)** Extraction of sheet resistance and contact resistance from the corresponding I-V data.

In order to explore sheet resistance and contact resistance of our designed β-Ga_2_O_3_ MOSFET, the I-V characteristics of CTLMs fabricated on β-Ga_2_O_3_ films after 5-minute and 7-minute F-plasma treatments were measured (**Fig. S1**). All I-V curves exhibit pronounced linear behavior, indicating that both 5-minute and 7-minute F-plasma treated CTLMs form ohmic contacts. The extracted sheet resistance ($R_{sh}$) are 53.5 MΩ/sq and 32.7 MΩ/sq, respectively. Correspondingly, the specific contact resistivity ($\rho_{c}$) are determined to be 20.6 Ω·cm^2^ and 23.1 Ω·cm^2^.


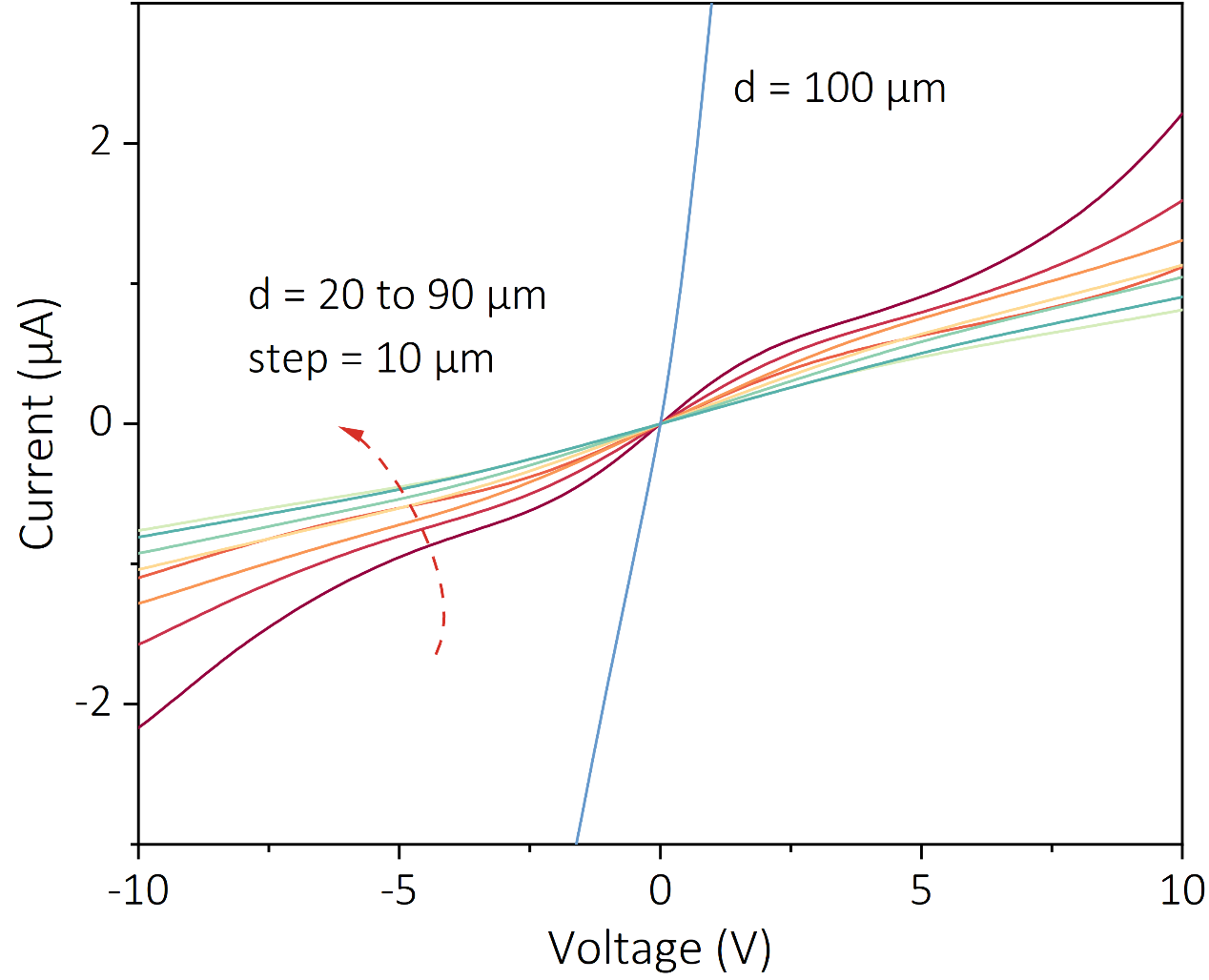
**Figure S2.** I-V curves of the CTLMs without F-plasma treatment.

**Fig. S2** presents the I-V curves of β-Ga_2_O_3_ CTLMs without F-based plasma treatment. The as-prepared β-Ga_2_O_3_ CTLMs exhibit nonlinear I-V characteristics, indicating the absence of satisfactory ohmic contact formation.


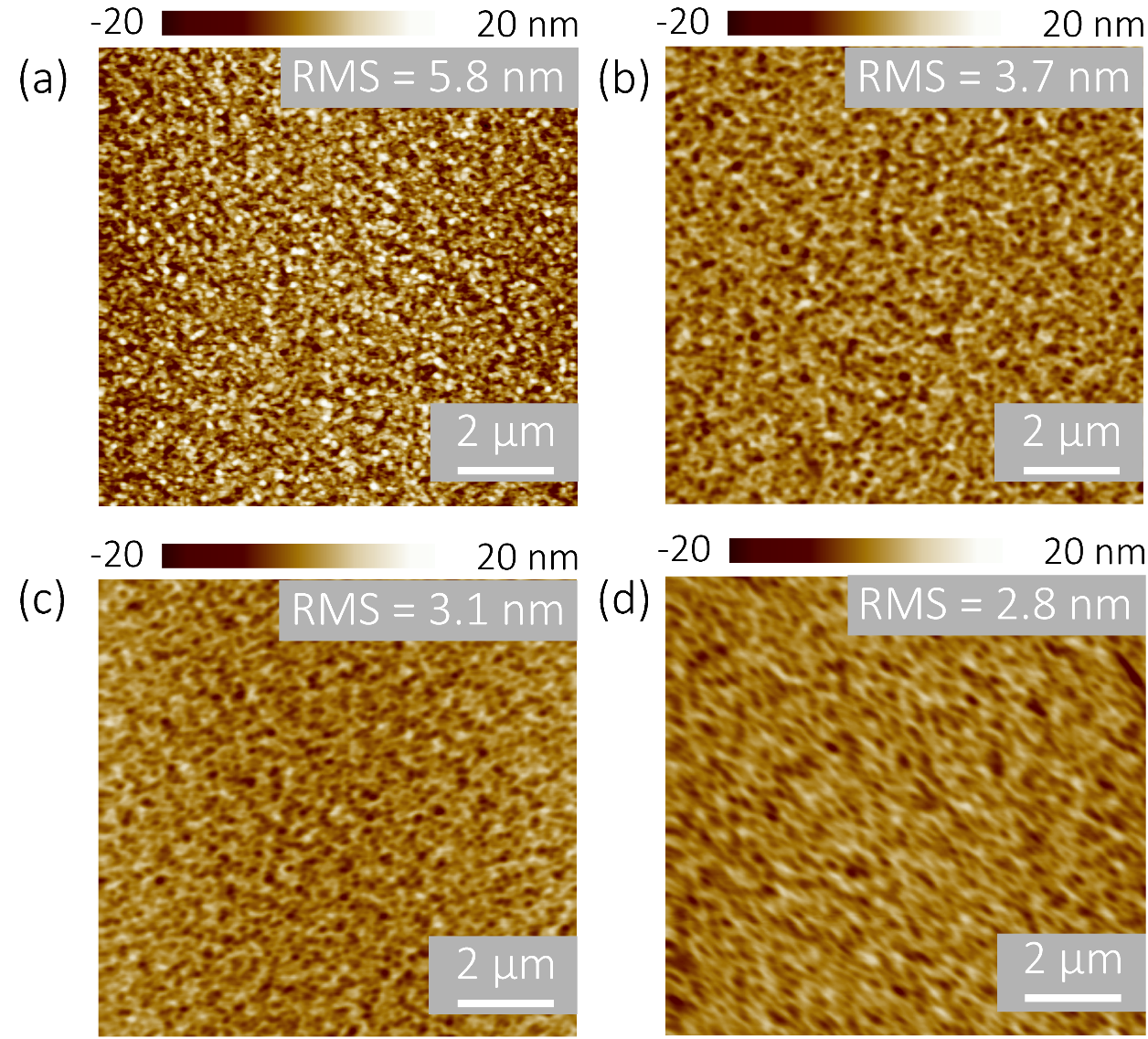


**Figure S3.** AFM images (10 × 10 μm^2^) and corresponding root-mean-square (RMS) surface roughness of β-Ga_2_O_3_ films: **(a)** as-grown, and after F-plasma treatment for **(b)** 3 min, **(c)** 5min, and **(d)** 7min.

To elucidate the influence of fluorine-based plasma exposure on surface morphology, AFM characterization was performed before and after treatment. As shown in **Fig. S3**, the β-Ga_2_O_3_ surface becomes progressively smoother with longer plasma exposure, as evidenced by the gradual reduction in RMS roughness. This trend indicates that the F-based plasma treatment effectively eliminates surface residues and modifies the near-surface microstructure, thereby improving the interfacial quality of both Ti/β-Ga_2_O_3_ and SiO_2_/β-Ga_2_O_3_ contacts. The smoother surface and cleaner interface are expected to suppress local field enhancement and defect-assisted tunneling, which can delay premature dielectric breakdown and contribute to the observed enhancement in breakdown voltage.


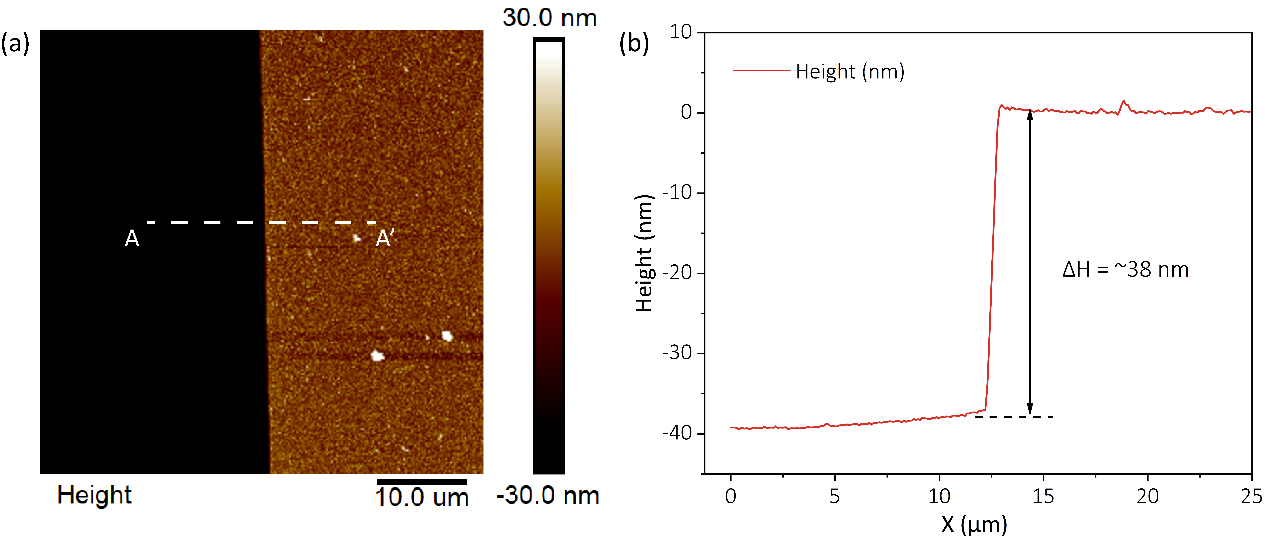


**Figure S4** **(a)** AFM image of the etched step height after 7 min F-plasma treatment using a photoresist mask. **(b)** Height profile extracted along the dashed line AA’ in **(a)**, showing a step height of approximately 38 nm, corresponding to an etch rate of ~5.4 nm/min.

To estimate the etch rate under our F-plasma conditions, the sample was subjected to the same plasma treatment using a photoresist mark for 7 minutes. The resulting step height was measured by AFM, as shown in **Figure S4**. The measured height of ~38 nm corresponds to an etch rate of approximately 5.4 nm/min. This etch rate is acceptable to the 300-nm thickness of our β-Ga_2_O_3_ thin films.


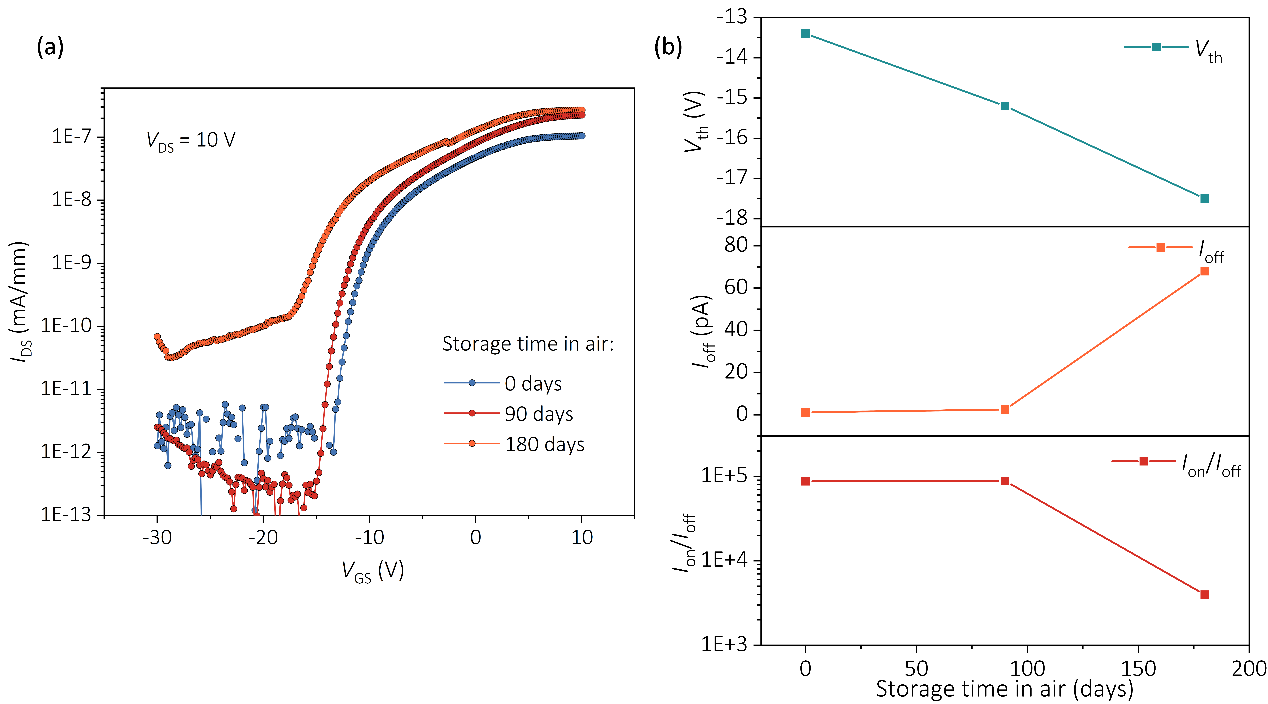


**Figure S5. (a)** Transfer characteristics of the β-Ga_2_O_3_ MOSFET (7-min F-plasma treated) measured after air exposure for 0, 90, and 180 days. **(b)** Extracted evolution of $V_{\mathrm{th}}$, $I_{\mathrm{off}}$, and $I_{\mathrm{on}}$/$I_{\mathrm{off}}$ as a function of storage time in air.

**Figure S5** illustrates the transfer characteristics of the 7-min fluorinated β-Ga_2_O_3_ MOSFET after storage in air for 0, 90, and 180 days. After 90 days of exposure, the device maintained stable electrical performance, with negligible changes in $I_{\mathrm{off}}$ and the $I_{\mathrm{on}}$/$I_{\mathrm{off}}$. The only noticeable shift is a moderate negative $V_{\mathrm{th}}$ shift of approximately –1.5 V. In contrast, after 180 days, clear degradation becomes evident: the $V_{\mathrm{th}}$ shift increased to about –3 V, the $I_{\mathrm{off}}$ rose by nearly two orders of magnitude, and the $I_{\mathrm{on}}$/$I_{\mathrm{off}}$ decreased to ~10³. These results indicate that prolonged ambient exposure gradually deteriorates the device characteristics, and further aging behavior beyond 180 days remains to be investigated.


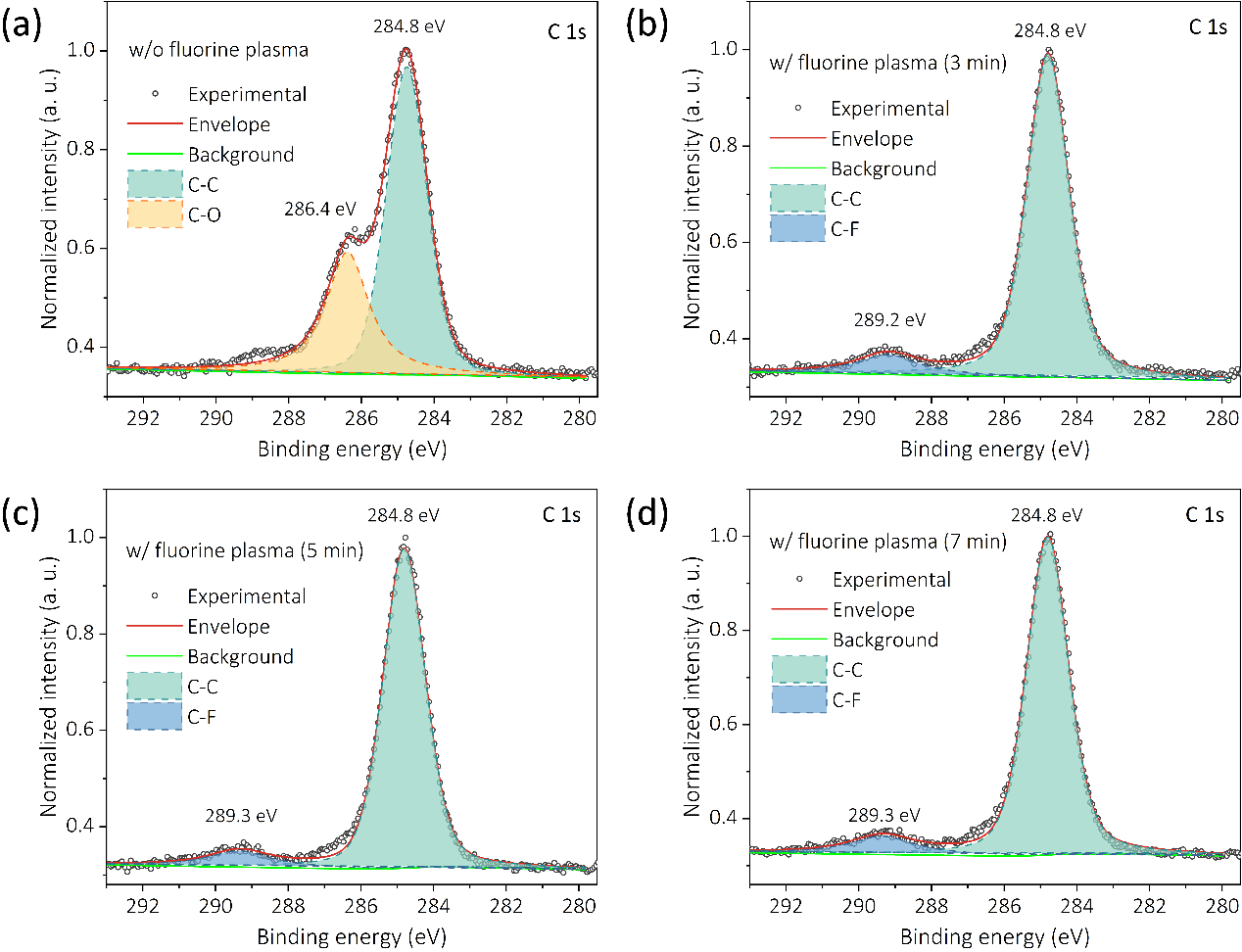


**Figure S6** C 1s XPS spectra for β-Ga_2_O_3_ films **(a)** without fluorine plasma, **(b)** with 3-minute, **(c)** 5 minute, **(d)** 7 minute fluorine plasma.

All XPS spectra were charge-corrected using the adventitious C 1s peak at 284.8 eV. As shown in **Figure S6**, the pristine sample exhibits two components at 284.8 eV (C–C) and 286.4 eV (C–O). After F-plasma treatment, the C–O signal disappears, and an additional peak emerges at 289.2 eV, which is consistent with the C–F bonding environment expected after CF₄/O₂ plasma exposure. This behavior confirms the presence of a small amount of C–F species on the plasma-treated surface.

**Table S1**. Summary of XPS fitting parameters (values from representative fits reported in the manuscript).

| Core level | Components | Peak position (eV) | FWHM (eV) |
| --- | --- | --- | --- |
| O 1s  w/o fluorine plasma | Ga-O | 530.6 | 1.3 |
|  | -OH | 532.3 | 1.6 |
| O 1s | Ga-O | 530.9 | 1.4 |
| w/ fluorine plasma (3min) | -OH | 532.2 | 1.8 |
| Ga 3d  w/o fluorine plasma | Ga^1+^ | 19.6 | 2.0 |
|  | Ga^3+^ | 20.1 | 1.2 |
| Ga 3d  w/ fluorine plasma (3min) | Ga^1+^ | 19.6 | 2.0 |
|  | Ga^3+^ | 20.4 | 1.4 |


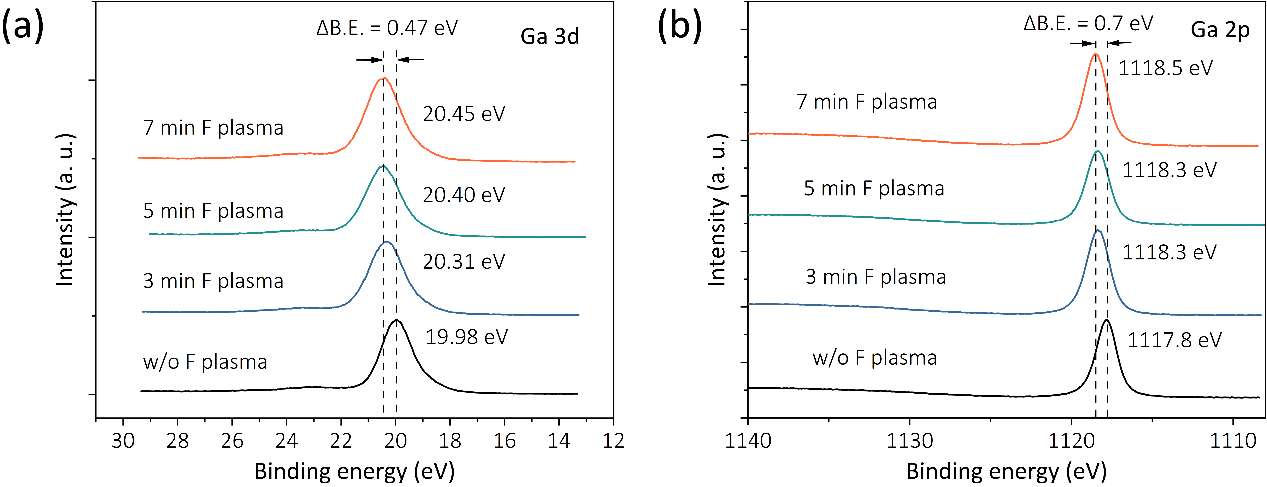


**Figure S7** **(a)** Overview of Ga 3d spectra and **(b)** Ga 2p for the untreated sample and samples subjected to 3, 5, and 7 min F-plasma treatments.

After F-treatment, both Ga 2p and Ga 3d exhibit chemical shifts toward higher binding energy, and their line shapes are well reproduced using a GL (Gaussian-Lorentzian) mixed profile, consistent with the behavior observed in the Ga 3d region. In addition, Ga 2p_1/2_ and Ga 2p_3/2_ peaks show a fixed spin-orbit splitting of 26.9 eV and an area ratio of approximately 0.49:1, which agrees with the expected splitting and intensity ratio of the Ga 2p doublet.


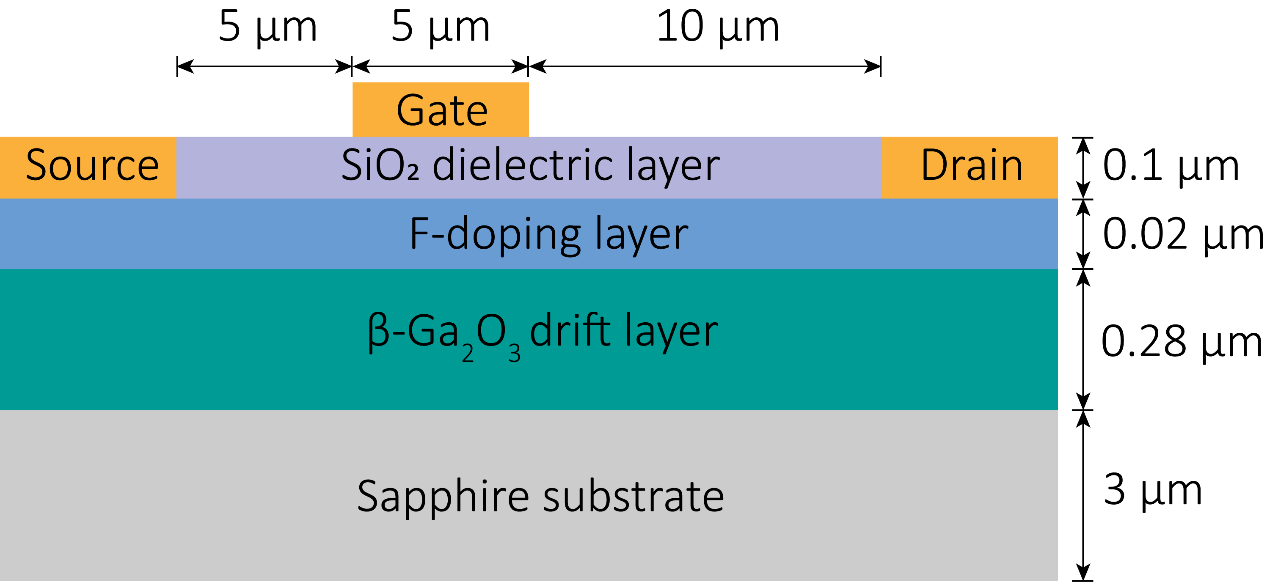


**Figure S8. Schematic cross-sectional view of the TCAD-simulated β-Ga_2_O_3_ MOSFET structure.**

Sentaurus TCAD simulations were conducted to investigate the charge-modulation behavior in β-Ga_2_O_3_ MOSFETs. The simulated device structure followed the experimental geometry, consisting of a SiO_2_ dielectric and a drift β-Ga_2_O_3_ layer. The key simulation parameters are summarized in **Fig. S8**. For computational efficiency and physical accuracy, the gate-to-source spacing ($L_{\mathrm{GS}}$), gate length ($L_{G}$) and gate-to-drain spacing ($L_{\mathrm{GD}}$) were proportionally scaled to 5 μm, 5 μm, and 10 μm, respectively.

Donor-like trap states associated with oxygen-vacancy-related defects were introduced into the β-Ga_2_O_3_ drift region. These traps were positioned approximately 1.0 eV below the conduction band minimum (CBM), consistent with previously reported defect levels in β-Ga_2_O_3_. To reproduce the experimentally observed near-surface defect distribution, the donor-like trap concentration ($N_{i}$) was qualitatively varied between the surface and bulk regions. Untreated devices were modeled with a higher near-surface trap concentration, whereas F-plasma-treated devices were assigned a reduced trap concentration in accordance with XPS-indicated passivation of oxygen vacancies.

It should be emphasized that the absolute numerical values used in the simulation were not intended to precisely quantify the defect densities. Instead, the TCAD model was designed to capture the qualitative trends—including defect neutralization, charge-density reduction, threshold-voltage modulation, and electric-field redistribution. Sensitivity analysis further confirmed the robustness of these trends, demonstrating that the observed physical behaviors do not strongly depend on the exact numerical choice of donor concentrations.


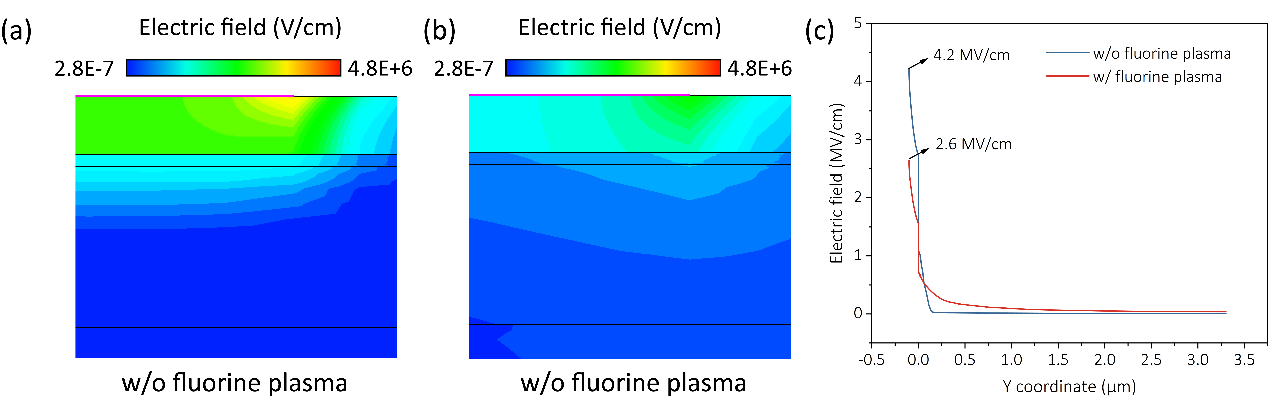


**Figure S9. (a), (b)** Simulated channel electric-field distributions of MOSFETs without and with F-treatment at $V_{\mathrm{GS}}$= -30 V and $V_{\mathrm{DS}}$= 10 V, respectively. **(c)** Electric-field profiles extracted along the dashed lines indicated in (a) and (b).

As shown in **Figure S9**, in the untreated MOSFET, the electric field is mainly concentrated in the SiO_2_ beneath the gate, with the peak field located near the gate edge on the drain side. After F treatment, the electric field redistributes and spreads downward into the Ga_2_O_3_ layer, alleviating field crowding and preventing premature gate breakdown, thereby enhancing the device’s breakdown voltage.
